# Presence of Circulating Anti-Myosin Antibodies in Endomyocardial Fibrosis

**DOI:** 10.1371/journal.pntd.0000661

**Published:** 2010-04-20

**Authors:** Ana Olga Mocumbi, Najma Latif, Magdi H. Yacoub

**Affiliations:** 1 Instituto do Coração, Maputo, Mozambique; 2 Heart Science Centre, Imperial College London, London, United Kingdom; 3 Magdi Yacoub Institute, London, United Kingdom; Emory University, United States of America

## Abstract

**Background:**

Endomyocardial Fibrosis (EMF) is a tropical restrictive cardiomyopathy of unknown etiology with high prevalence in Sub-Saharan Africa, for which it is unclear whether the primary target of injury is the endocardial endothelium, the subendocardial fibroblast, the coronary microcirculation or the myocyte. In an attempt to explore the possibility of endocardial lesions being a result of an immune response against the myocyte we assessed the presence and frequency of circulating anti-myocardial antibodies in EMF patients.

**Methodology/Principal Findings:**

EMF classification, assessment of severity and staging was based on echocardiography. We used sodium dodecylsulfate polyacrylamide gel electrophoresis (SDS-PAGE) of myocardial proteins followed by western blotting to screen serum samples for antiheart antibodies G and M classes. The degree of serum reactivity was correlated with the severity and activity of EMF. We studied 56 EMF patients and 10 healthy controls. IgG reactivity against myocardial proteins was stronger and more frequent in patients with EMF when compared to controls (30/56; 53.6% vs. 1/10; 10%, respectively). IgM reactivity was weak in both groups, although higher in EMF patients (11/56; 19.6%) when compared to controls (n = 0). EMF patients showed greater frequency and reactivity of IgG antibodies against myocardial proteins of molecular weights 35 kD, 42 kD and 70 kD (*p* values <0.01, <0.01 and <0.05 respectively).

**Conclusions:**

The presence of antibodies against myocardial proteins was demonstrated in a subset of EMF patients. These immune markers seem to be related with activity and might provide an adjunct tool for diagnosis and classification of EMF, therefore improving its management by identifying patients who may benefit from immunosuppressive therapy. Further research is needed to clarify the role of autoimmunity in the pathogenesis of EMF.

## Author Summary

Endomyocardial Fibrosis is a tropical disease in which the heart cannot open properly to receive blood due to a scar that covers its inner layer. It affects mainly children and adolescents, and has a poor prognosis because the cause and mechanisms of scarring are unknown. The conventional treatment is frustrating and does not alter the natural history of the disease. Despite affecting several million people worldwide there has been little investigation on the mechanisms of the disease or drug development to improve its prognosis. In this study we investigate the presence of antibodies against the myocardial cells of African patients with severe and advanced EMF aiming at uncovering new pathways for the disease. Our results reveal that EMF patients have anti-myocardial antibodies in their blood. The reaction of these antibodies with the heart may be one of the mechanisms involved in the genesis of the fibrotic lesions. This knowledge may help in diagnosing the condition and provide alternatives for its management, using drugs that reduce the impact of the circulating antibodies in the cardiac tissue. The significance of these results needs confirmation on studies involving larger number of subjects due to frequent finding of antiheart antibodies in African populations with heart failure of any cause.

## Introduction

Endomyocardial Fibrosis (EMF) is a tropical cardiomyopathy of unclear etiopathogenesis and poor prognosis, which is endemic in certain regions of sub-Saharan Africa [1]. It is probably the commonest form of restrictive cardiomyopathy, affecting primarily children and adolescents. The distinctive pathological feature of established EMF is endocardial thickening of one or both ventricles, more prominent at the apices and the inflow tracts, usually causing dysfunction of the atrioventricular valve [1], [2].

The diagnosis of EMF is usually made in late stages of the disease, when heart failure or its complications are already present, and is based on clinical and echocardiographic features. Although hypereosinophilia is a common finding in African patients, no biological marker is currently available for early detection. Medical management of EMF aims at controlling episodes of heart failure and its complications, as well as treating hypereosinophilia using oral corticosteroids [2], [3]. Surgery is recommended to symptomatic patients since it increases survival [4] and improves the quality of life, but has been associated with high morbidity and mortality [5], and has progressed slowly due to lack of facilities for open-heart surgery in most regions where the disease is endemic.

The primary target of injury in EMF is not known. It has been suggested that the endomyocardial lesions may be the result of a primary injury to the endocardial endothelium, subendocardial fibroblast, coronary microcirculation or myocytes [3].

In an attempt to explore the possibility of endocardial lesions being a result of an autoimmune response against the myocytes we assessed the presence and frequency of circulating IgM and IgG class anti-myocardial antibodies in different forms and stages of the disease.

## Methods

Serum was obtained from 56 consecutive EMF patients from the Mozambican clinical registry and 10 blood donors from the same population. All controls were submitted to transthoracic echocardiography to rule out the presence of cardiac disease.

### Ethics *s*tatement

The National Bioethical Committee for Health from Mozambique approved the study protocol. Written informed consent was obtained from all patients and controls.

### Protocol for clinical evaluation of patients

EMF diagnosis was based on the demonstration of mural and/or valvar endocardial thickening and other echocardiographic features of EMF described elsewhere [2]. The disease was defined as right (REFM), left (LEMF) and bilateral (BEMF) according to the predominance of structural lesions in one or both sides of the heart. The severity of endocardial lesions was determined using a standardized scoring system that defines four different grades, namely mild (I), moderate (II), severe (III) and advanced (IV) [6]. Finally, activity was defined based on the presence of clinical sings such as fever, periorbital edema, urticaria, recrudescence of heart failure, and laboratory findings of increased erythrosedimentation rate and severe hypereosinophilia (absolute eosinophil count >1.5×10^9^/L); in the absence of any of those clinical sings for more than 6 months patients were considered to have remission (quiescent disease) [7].

### SDS-PAGE and Western blotting

Normal ventricular myocardium was obtained from a donor heart and immediately frozen in liquid nitrogen. The myocardial samples were pulverized while still frozen and homogenized in 1% SDS. A protein assay was carried out using the dye-binding procedure of Bradford. Samples (25 µg) were solubilised and denatured by heating at 70°C for 10 minutes in LDS sample buffer (Invitrogen). The samples were loaded onto 10% tris-bis gels and run at 60 mA/gel until the tracking dye reached the end of the gel. Molecular weight markers (Amersham) were concomitantly run on each gel. The proteins were electrophoretically transferred to nitrocellulose at 30 V for 1 hour.

### Detection of antiheart antibodies

Nitrocellulose strips carrying separated lanes of myocardial proteins separated by SDS-PAGE were blocked for 1 h with 3% w/v nonfat dried milk (Marvel) in phosphate buffered saline containing 0.05% w/v Tween-20 (blocking solution). Strips were then incubated with patient's serum, diluted 1∶100 in blocking solution, and agitated for 1 h at room temperature. After thorough washing in PBS-twin strips were incubated for a further 1 h in either peroxidase-conjugated rabbit antihuman IgG (Dako) or IgM (Dako) at a dilution of 1∶500 in blocking solution. The strips were then washed thoroughly in PBS. Protein bands to which antiheart antibodies had bound were visualized using enhanced chemiluminescence detection system (ECL, Amersham). Blots were incubated with the detection reagent for 1 min and then exposed to Hyperfilm (Amersham) for 1, 2 and 5 minutes. The films were developed using an automated radiograph developer.

We defined strong reactivity to either IgG or IgM as the presence of five or more antiheart antibodies of each class.

### Data analysis

Quantitative data are given as means ± SD. Fischer exact test was used to compare the positive results in patients and controls. Statistical significance was defined as p values <0.05. T-test and analysis of variance were used to compare the differences between groups of patients divided by type, severity and disease activity.

## Results

### Characterization of patients and controls

**Figure 1:**
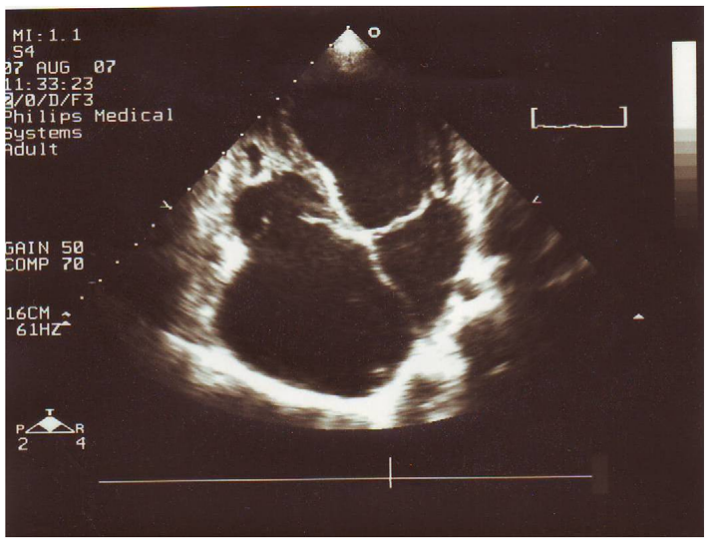
Severe right endomyocardial fibrosis. Echocardiography usually reveals partial obliteration of the right ventricle with cavity reduction, marked thickening of the moderate band, right atrial and tricuspid annulus dilatation, associated severe with tricuspid regurgitation. Although there is thickening of both leaflets of the atrioventricular valves, no endocardial thickening is seen on the left side of heart.

**Figure 2:**
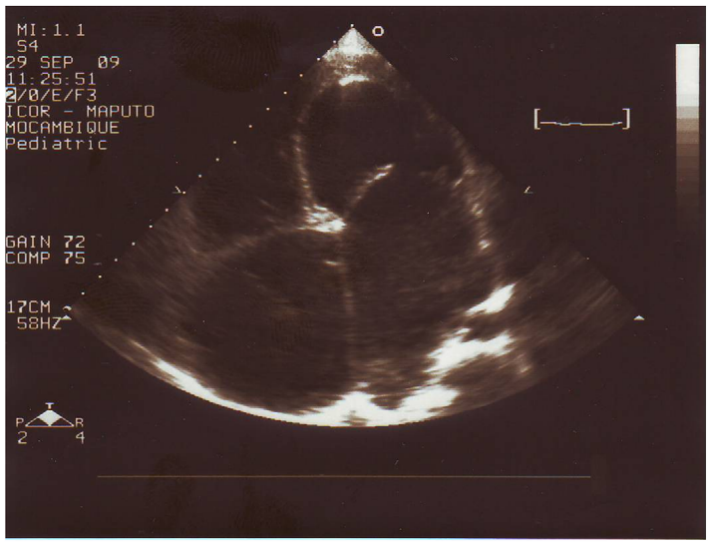
Severe left endomyocardial fibrosis. Echocardiography shows thickening of endocardium at the apex of the left ventricle that has a spherical shape and has reduced longitudinal dimension and thickening of the anterior leaflet of the mitral valve; left atrial dilatation is associated with both mitral regurgitation and reduced ventricular compliance. Notice the dilatation of the right cavities caused by elevation in pulmonary pressures.

The mean age of patients was 18±11 years and there were 35 females (62.5%), while the controls had a mean age of 20±2 years and 5 (50%) were females. Forty-five patients (80.3%) were in New York Heart Association functional classes III/IV and 18 (32.1%) had atrial fibrillation. Thirty-two patients (57.1%) had BEMF, 20 (35.8%) had REMF (**Figure 1**) and 4 (7.1%) had LEMF (**Figure 2**). Advanced (grade IV) disease was present in 35 (62.5%) patients. Severe hypereosinophilia was presented in 12 (21.4%) patients; 8 (14.3%) had active disease. **Table 1** presents patient's characteristics.

**Table 1:** Characteristics of the 56 patients with endomyocardial fibrosis (EMF).

Variable	Value (%)
Age (years)	18±11
Male/Female	21/35
NYHA class I	8 (14.3)
II	3 (5.4)
III	22 (39.3)
IV	23 (41.0)
Severity of EMF Mild	13 (23.2)
Moderate	8 (14.3)
Severe & Advanced	35 (62.5)
Active disease	8 (14.3)
Atrial fibrillation	18 (32.1)

### Detection of IgG and IgM

Fifty-four (96.4%) patients tested positive for IgG class anti-myocardial antibodies whilst 33 (58.9%) tested positive for those of IgM class. A higher frequency of IgG class antibodies was found in EMF patients when compared with healthy controls. The difference was statistically significant for proteins of the following molecular weights: 42 kD (33; 58.9% in patients versus 0 in controls; *p* = 0.0009), 35 kD (32; 57.1% in patients vs 0 in controls; *p* = 0.0009) and 70 kD (23; 41.1% in patients versus 0 in controls; p = 0.011). Patients had also higher frequencies of antiheart antibodies against proteins of 60 kD (18; 32.1% versus 0) and 90 kD (19; 33.9% versus 0) molecular weights, although the differences were not statistically significant (*p* values of 0.051 and 0.052, respectively). The summary of frequencies of IgG class anti-myocardial antibodies in patients with EMF and control subjects is presented in **Table 2**.

**Table 2:** Frequency of IgG antiheart antibodies in patients with endomyocardial fibrosis (EMF) and healthy controls.

Protein	EMF		Controls		
Molecular Weight	______ 56 ______		______ 10 ______		Fisher Exact Test
(kD)	N+ (%)	N–	N+ (%)	N–	
25	13 (23.2)	43	1 (10)	9	0.68
30	13 (23.2)	43	0 (0)	10	0.19
35	32 (57.1)	24	0 (0)	10	0.0009
42	33 (58.9)	23	0 (0)	10	0.0009
46	21 (37.5)	35	0 (0)	10	0.024
60	18 (32.1)	38	0 (0)	10	0.051
70	23 (41.1)	33	0 (0)	10	0.011
80	24 (42.9)	32	1 (10)	9	0.076
90	19 (33.9)	37	0 (0)	10	0.052
100	20 (35.7)	36	2 (20)	8	0.48
110	10 (17.9)	46	0 (0)	10	0.34
150	15 (26.8)	41	2 (20)	8	1.00

N+ number testing positive; N- number testing negative; kD kilo Daltons.

Controls were negative for all IgM class antibodies but the difference in frequencies between EMF patients and control subjects was not statistically significant for any protein. The proteins more frequently detected were 46 kD (17; 30.4% versus none in controls, *p* = 0.053) and 80 kD (12; 21.4% versus none in controls, *p* = 0.19). **Table 3** shows the frequency of anti-myocardial antibodies of IgM class in patients with endomyocardial fibrosis and control subjects.

**Table 3:** Frequency of IgM antiheart antibodies in patients with endomyocardial fibrosis and healthy controls.

Protein	EMF		Controls		
Molecular Weight	______ 56 ______		______ 10 ______		Fisher Exact Test
(kD)	N+ (%)	N –	N+ (%)*	N –	
25	6 (10.7)	50	0	10	0.58
35	8 (14.3)	48	0	10	0.34
40	6 (10.7)	50	0	10	0.58
42	9 (16.1)	47	0	10	0.33
44	6 (10.7)	50	0	10	0.58
46	17 (30.4)	39	0	10	0.053
50	11 (19.6)	45	0	10	0.19
60	11 (19.6)	45	0	10	0.19
70	7 (12.5)	49	0	10	0.58
80	12 (21.4)	44	0	10	0.19
90	4 (7.1)	52	0	10	1.00
100	6 (10.7)	50	0	10	0.58

* All percentages are equivalent to zero.

N+ number testing positive; N- number testing negative; kD kilo Daltons.

### Reactivity according to disease type, activity and severity

The mean number of antibodies was higher for LEMF (14.0±9.1), than for BEMF (8.5±6.0) and REMF (8.4±4.2), but only 4 patients with LEMF were positive for anti-myosin antibodies. No significant difference was found in the mean number of antibodies in these three groups (*p* = 0.18, ANOVA).

There were 35 patients with severe and advanced EMF; their mean number of antibodies (9±5) was not statistically different from that found in patients with grades I/II (9±7; *p* = 0.68, t-test).

**Table 4** shows the characteristics of the 8 patients with active disease; 4 had BEMF, 2 REMF and the remaining 2 had LEMF. The mean number of antibodies detected was greater for patients with disease (19.6±3.7) when compared to those with quiescent disease (7.1±3.3) with a *p* value <0.001.

**Table 4:** Characteristics of the eight patients with active EMF.

Code	P1	P2	P3	P4	P5	P6	P7	P8
**EMF type**	LEMF	LEMF	BEMF	BEMF	BEMF	BEMF	REMF	REMF
**Number IgG**	10	14	14	10	11	11	9	6
**Number IgM**	6	8	0	10	9	7	4	12
**Total**	16	22	14	20	20	18	13	18

P patient.

LEMF left EMF; REMF right EMF; BEMF bilateral EMF.

I  =  mild; II  =  moderate; III  =  severe & advanced.

## Discussion

Autoantibodies against myocardial proteins were detected in EMF patients, suggesting a role of autoimmunity in a subset of patients with this cardiomyopathy. These data corroborate previous findings from Nigeria [8] and India [9], [10], and have the advantage of being obtained using a technique whose results are easier to interpret [11]. They also support the need to evaluate the role of immunosuppressive therapy in the management of EMF, considering that its current treatment is deceiving and does not alter natural history.

Better understanding of the significance of antiheart antibodies in cardiac disease is needed since they have been demonstrated in patients with and without heart disease in areas of Africa endemic for EMF [12], [13], [14], and occur commonly after several types of cardiac injury such as surgery [15], [16] and myocardial disease [17] suggesting a non-pathogenic role. Although there has been failure to demonstrate their *in vitro* and *in vivo* cytotoxicity [18], a cause-and-effect relationship between circulating cardiac myosin autoantibodies and impairment of myocyte contractility was recently demonstrated [19]. Moreover, circumstantial evidence implicates heart-reactive antibodies in forms of heart disease such as Rheumatic Heart Disease [20], [21], cardiomyopathies [22], post-myocardial infarction (Dressler's) syndrome, post-pericardiotomy syndrome [15], and rejection events following transplantation [23], and immunosuppressive therapy decreases the levels of such antibodies improving the clinical condition.

It has been suggested that antiheart antibodies are a non-invasive marker of early disease in some forms of cardiomyopathy [23], binding to specific cardiac tissue later on, hence the reduction in circulating levels in advanced forms of dilated cardiomyopathy [22], [24]. We could not replicate these findings in EMF patients as those with advanced disease presented stronger reactivity and greater number of anti-myosin antibodies.

Antiheart antibodies were predominant in patients with active disease, defined by the presence of clinical and/or laboratory signs of inflammation, including hypereosinophilia. Future research must clarify the role of hypereosinophilia and define the temporal changes in autoimmune response in earlier phases of EMF to allow the use of autoantibodies as a diagnostic tool.

There was high frequency of antibodies of molecular weights of 42 kD, 35 kD and 70 kD, corresponding to Actine, Tropomyosin and Heat Shock Protein-70 (HSP-70), respectively. HSP-70 is an extracellular stress protein that can function as a potent immunological adjuvant attenuating the inflammatory disease via apparent effects on immunoregulatory T cell populations [25]. The presence of antibodies to this protein in 41% of our patients may indicate that an additional source of injury in EMF might be the reduction of proteins involved in cytoprotection.

Our results must be interpreted with caution since lesions of the endocardium, the myocardium and the interstitium occur in EMF patients. The homogenized piece of myocardium contains not only myocardial fibers but also elements of the interstitium and blood vessel wall, indicating that further research is needed aiming at identification of the exact proteins targeted by these autoantibodies. Most patients had severe heart failure, which usually correlates to seropositivity to autoantibodies, irrespective of the nature of the heart condition [13]. The fact that the majority of our patients were females (62.5%) might have influenced the results since there is higher prevalence of autoimmune disease in women, for reasons that are incompletely understood [26]. Another factor that must be taken into consideration is the possibility of cardiac, skeletal and smooth muscle sharing some antigenic determinants, in which case the antibodies would not be entirely organ specific. Since hypereosinophilia and recrudescence of heart failure were criteria for definition of heart failure, it cannot be excluded that part of the hyperreactivity was only a response to these situations. Finally, the finding of immune markers in only a proportion of EMF patients suggests that endomyocardial damage is also mediated by mechanisms other than autoimmunity.

### Conclusion

High levels of circulating anti-myosin antibodies are present in a subset of patients with EMF. Although their role remains unclear, these autoimmune markers may provide an adjunct tool for the classification of EMF and improve its management, by identifying patients who could benefit from immunosuppressive therapy. Efforts must be made to clarify the role of autoimmunity in the pathogenesis of EMF.

## Supporting Information

Alternative Language Abstract S1 Translation of the abstract into Portuguese by AOM.
(0.02 MB DOC)
