# Supplementary material for: Presence of Circulating Anti-Myosin Antibodies in Endomyocardial Fibrosis
Source: PLoS Negl Trop Dis. 2010 Apr 20;4(4):e661. doi: 10.1371/journal.pntd.0000661 (PMC2857887; doi:10.1371/journal.pntd.0000661)
Supplement: Alternative Language Abstract S1 — Translation of the abstract into Portuguese by AOM. (0.02 MB DOC) [file pntd.0000661.s001.doc]

**Resumo**

**Introdução**: A fibrose endomiocárdica (FEM) é uma miocardiopatia restrictive tropical de causa desconhecida com elevada prevalência na África Sub-Sahariana, para a qual se desconhece se o alvo primário de lesão é a endotélio endocárdico, o fibroblasto subendocárdico, a circulação corornária ou a célula miocárdica. Com o objectivo de explorar a possibilidade das lesões endocárdicas resultarem duma resposta imunitária dirigida ao miócito foi investigada a presença e frequência de anticorpos anti-miocárdicos circulantes em pacientes com FEM.

**Metodologia e Achados Principais:** A classificação, definição da severidade e estadiamento da FEM realizou-se por ecocardiografia transtorácica. Foi usada a técnica de *sodium dodecylsulfate polyacrylamide gel electrophoresis (SDS-PAGE)* de proteínas miocárdicas seguida de *western blotting* para identificar anticorpos antimiocárdicos de classes G e M no soro. O grau de reactividade do soro foi correlacionado com a severidade e actividade da FEM. Foram estudados 56 doentes com FEM e 10 controles saudáveis. A reactividade IgG contra proteinas miocárdicas foi mais forte e mais frequente em doentes em comparação com os controles (30/56; 53.6% versus 1/10; 10%, respectivamente). Os pacientes mostraram grande frequência e reactividade de anticorpos IgG contra proteínas miocárdicas de pesos moleculares de 35kD, 42kD e 70kD (valores de *p* <0.01, <0.01 e <0.05 respectivamente). A reactividade IgM foi nula nos controles e fraca nos pacientes (11/56; 19.6%).

**Conclusões:** Demonstrou-se a presença de anticorpos antimiocárdicos em paret dos pacientes com FEM. Estes marcadores imunológicos parecem estar relacionados com doença activca e têm potencial para ser usados como um critério adicional no diagnóstico e classificação da FEM, podendo ainda melhorar o tratamento da doença através da identificação de pacientes que podem beneficiar de tratamento imunossupressor. É necessária investigação para clarificar o papel da autoimunidade na patogénese da FEM.
